# Case report: rare presentation of primary angiomyolipoma in the middle ear

**DOI:** 10.1186/s12893-020-00956-9

**Published:** 2020-11-18

**Authors:** Fei Wang, Hao Wang

**Affiliations:** 1 Department of Otolaryngology Head and Neck Surgery, People’s Hospital of Qinghai Province, Xining, China; 2 Department of Intensive Care Unit, People’s Hospital of Qinghai Province, Xining, China

**Keywords:** Middle ear angiomyolipoma, Diagnosis, Treatment

## Abstract

**Background:**

Angiomyolipoma (AML) is a common, chronic disease usually localized in kidney and liver organs; but occurring frequently outside the kidney or liver.

**Case presentation:**

We describe an unusual case of a 62-year-old female patient with AML in the middle ear. She presented with left earache, tinnitus and hearing loss. Preoperative computed tomography and magnetic resonance imaging seemed to reveal a middle ear cholesteatoma. The patient underwent surgical resection of this lesion, and the AML was finally confirmed by histopathological examination. The patient was discharged on the 8th postoperative day and did not seek further treatment.

**Conclusions:**

Extraperitoneal AML is rare and accurately identified by histopathology. The recommended management is surgery for AML in the middle ear.

## Background

Angiomyolipoma (AML) is the most common benign tumour of the kidney and is related to the tuberous sclerosis complex, which is composed of vascular endothelial cells, smooth muscle cells and fat cells [1–3]. Patients with tuberous sclerosis complexes often have multiple renal AMLs [4]. Additionally, AML is frequently found among women with lymphangioleiomyomatosis [5]. Both incidental and disease-related AML are caused by mutations in the TSC1 or TSC2 genes that control cell growth and proliferation [6]. However, this tumour outside the kidney is extremely rare. Given the paucity of information about this tumour, the aim of this study was to present a case, the first AML reported in the middle ear, to describe the clinical features and a treatment protocol.

## Case presentation

A 62-year-old female patient was admitted to the hospital in July 2019 because of left earache, tinnitus, hearing loss for 30 years and worsening earache for more than 1 month. She had not received any treatment before. The patient's personal history, past history and family history were unremarkable.

**Fig. 1:**
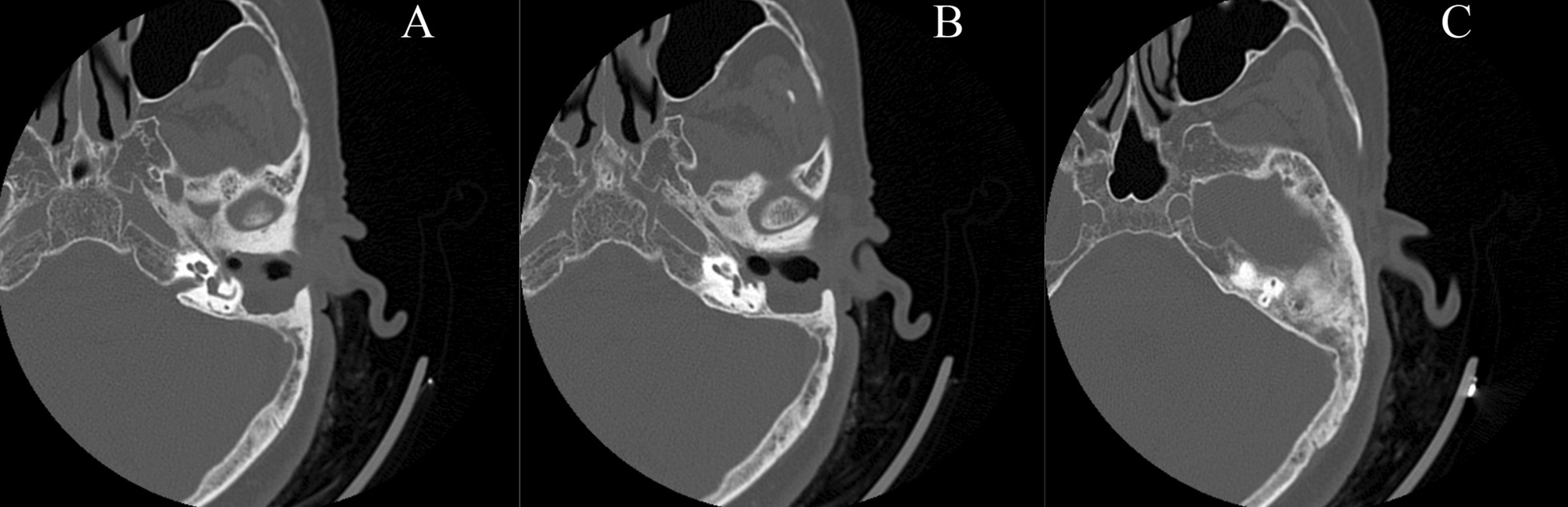
Computed tomography (**a**, **b**, **c**) revealed part of the left temporal bone

**Fig. 2:**
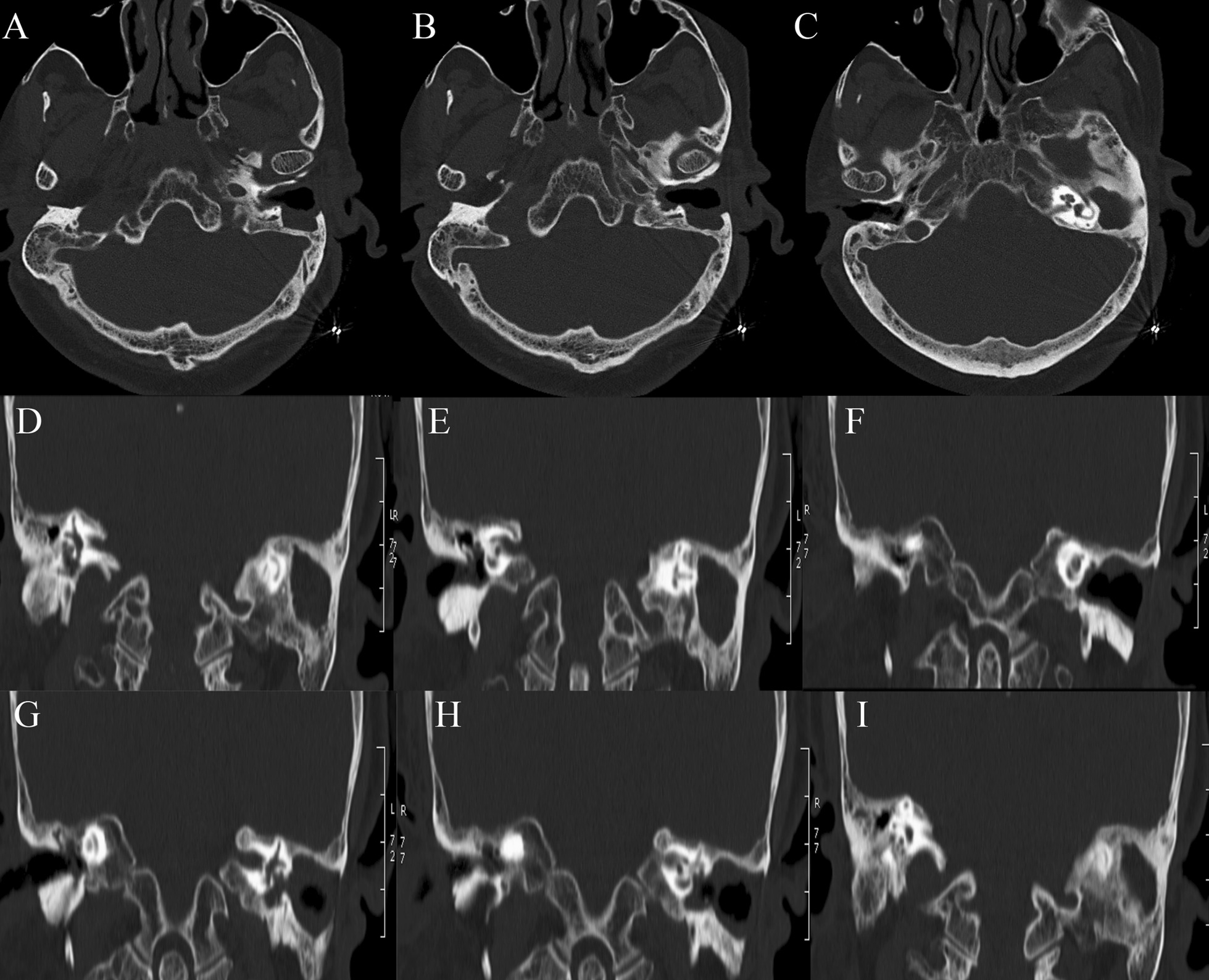
Computed tomography appearance in axial imagines (**a**, **b**, **c**)) and coronal imagines (**d**, **e**, **f**, **g**, **h**, **i**)

**Fig. 3:**
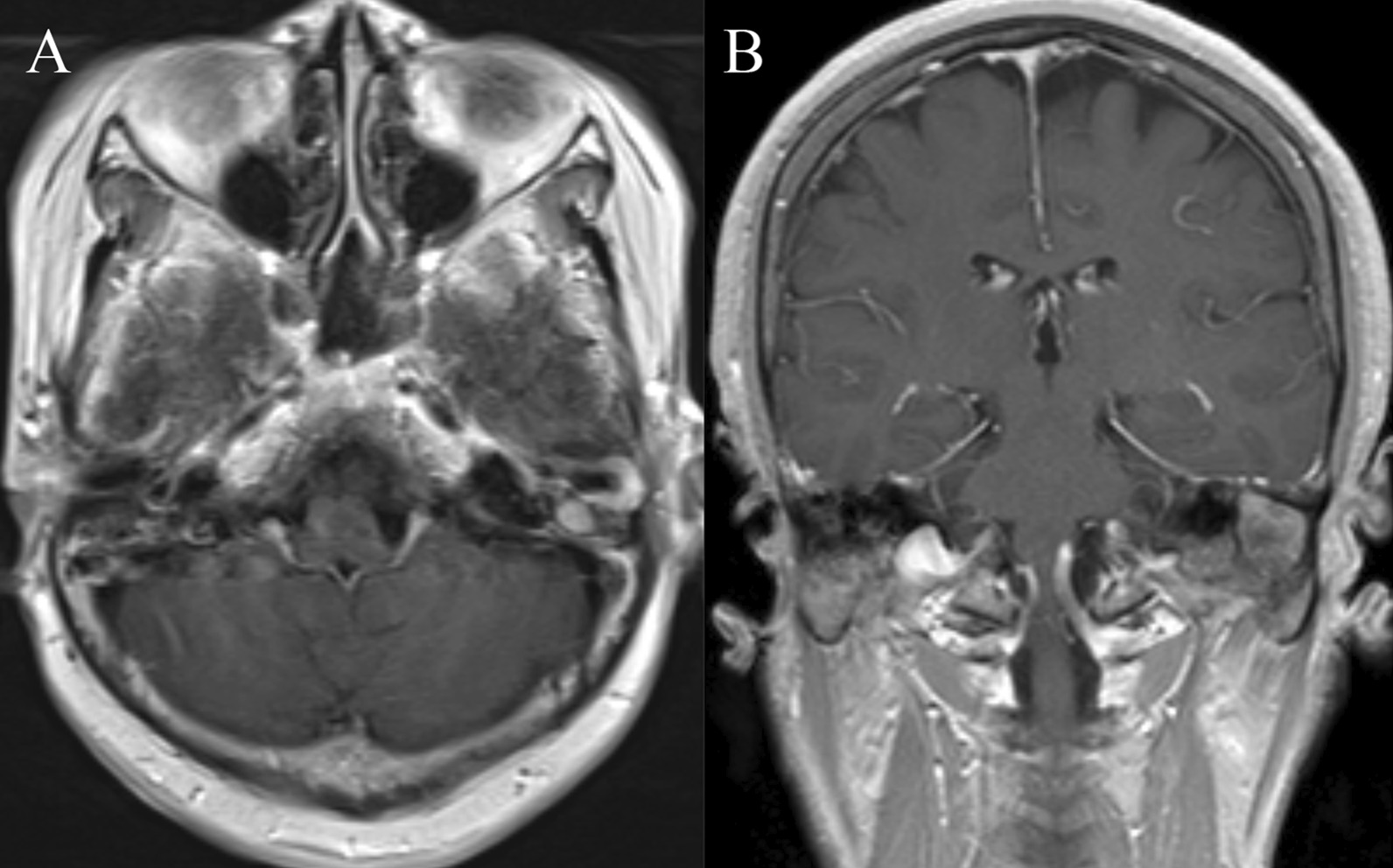
Magnetic resonance imaging revealed structures of the tumour

**Fig. 4:**
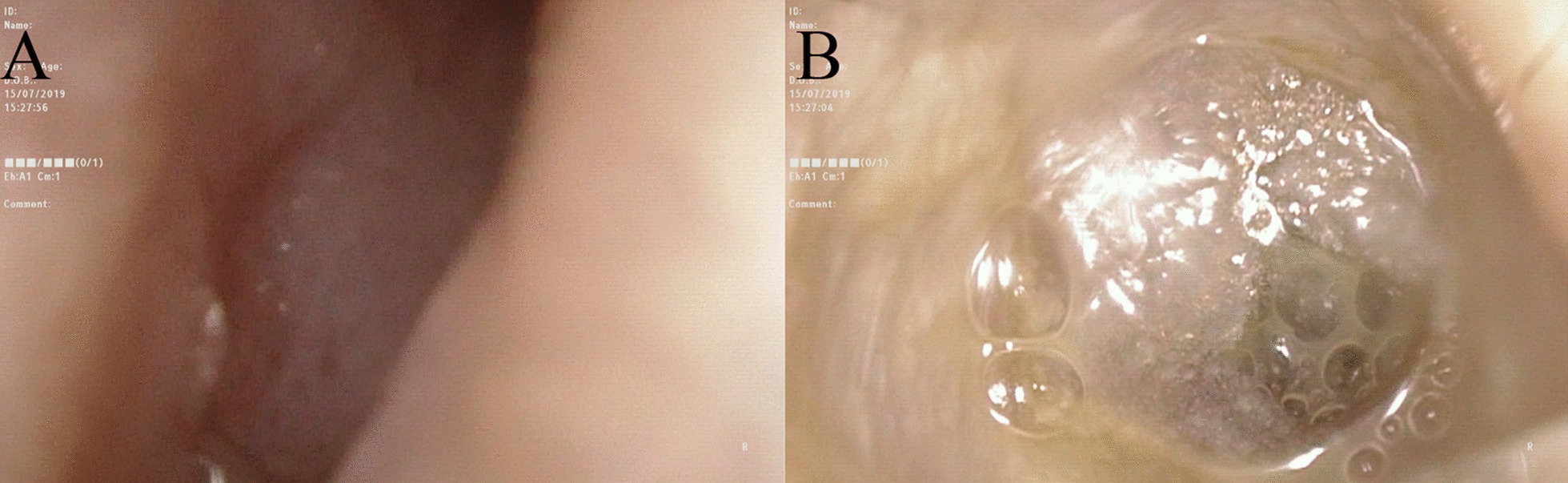
Otoscope **a** (left) and **b** (right) showed external auditory meatus and the tympanic membrane

**Fig. 5:**
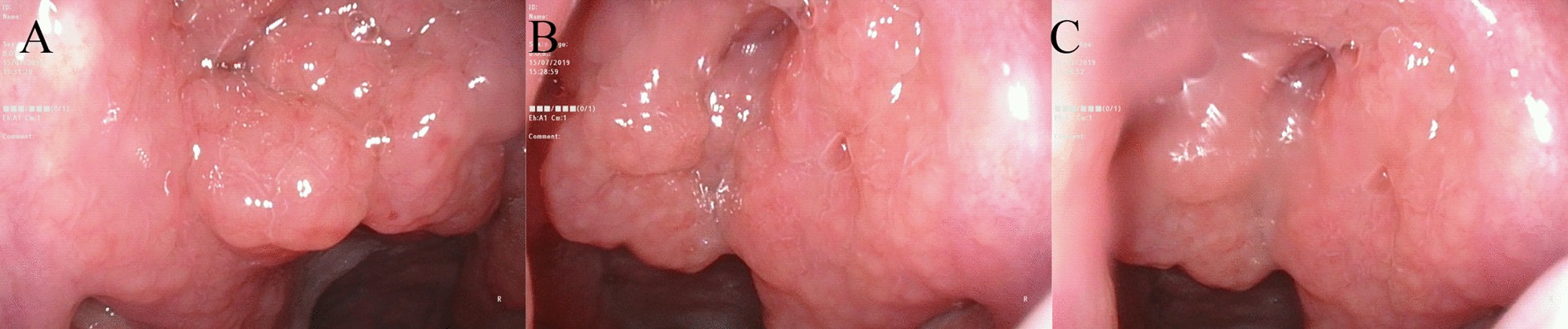
Nasopharynx showed adenoid hyperplasia and hypertrophy

Renal ultrasound, lung computed tomography (CT) and skin examination were performed in this patient to preclude the potential AML of other parts of the body outside the middle ear. On physical examination, the bilateral auricles showed no deformity or traction pain, and a large amount of purulent secretions were found in the left external auditory canal. Cerumen debris was found in the right external auditory canal, and no obvious tenderness was found in the bilateral mastoid process area. The rinne test results were as follows: left (+), right (−). The weber test result was right-sided (+). Temporal bone CT showed right otitis media mastoiditis, partial defect of the left temporal bone, no normal structure in the middle ear cavity and soft tissue thickening in the left external auditory canal (Fig. 1). To determine the extent to which the surrounding structures were destroyed by the tumour, especially any damage to the tympanic wall, axial and coronal CT was further performed and indicated a partial defect in the left temporal bone, no normal structure in the middle ear cavity and soft tissue thickening of the left external auditory canal (Fig. 2). Cranial enhanced magnetic resonance imaging (MRI) suggested an abnormal signal in the left middle ear: suspected cholesteatoma (Fig. 3). Oto-endoscopy suggested that the orifice of the left ear was narrow with a large number of secretions in the outer ear canal, and the tympanic membrane could not be seen; the tympanic membrane of the right ear was intact, vacuolar, and indistinct (Fig. 4). Nasopharyngoscopy suggested adenoid hyperplasia (Fig. 5). Pure tone audiometry suggested sensorineural deafness in the left ear and an average threshold of air conduction of 103 dB and conductive deafness in the right ear and a mean bone conduction threshold of 32 dB and a mean air conduction threshold of 62 dB.

**Fig. 6:**
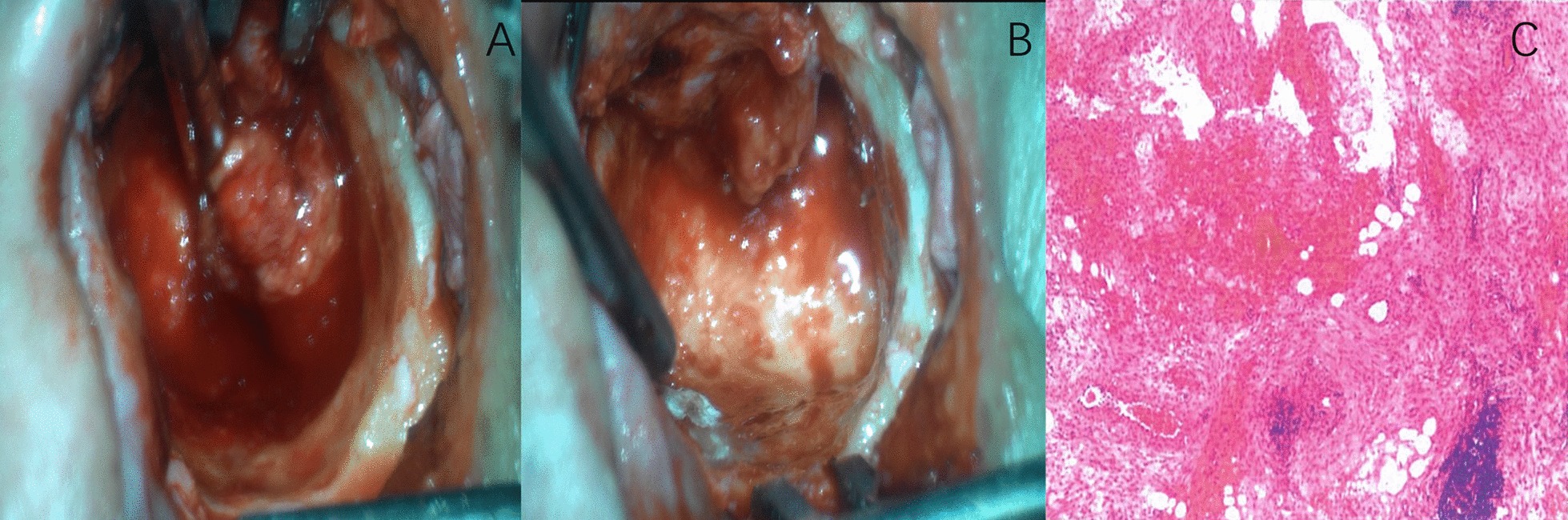
**a** and **b** Intraoperative progress; **c** pathology

The operation was performed under general anaesthesia on the 7th day after admission. After general anaesthesia took effect, the patient was placed in a horizontal position, his head turned to the right, and the affected ear was upward. Ten millilitres of normal saline, 8 drops of pararenin and 10 ml ropivacaine were used for periauricular anaesthesia and haemostasis. The tympanic sinus was opened from the ethmoidal region, and the lateral wall of the superior tympanic cavity and the residual posterior wall of the auditory canal were removed. The facial nerve crest was ground down. The superior tympanic chamber, posterior tympanic chamber and mastoid were fused into a large cavity full of granulation. The granulation was carefully peeled off, and the facial nerve was explored. The bone canal was intact, the semicircular canal bone was not damaged, and most of the malleus and incus were rotten and lost. The residual ossicles were removed, and no residual granulation was found in the tympanum. The operation cavity was washed with normal saline, and the flap of the external auditory canal was cut and smoothed back. The operation cavity was filled with a gelatine sponge, and the incision was sutured. The granulation was sent to pathology. Intraoperatively, we observed that the mastoid bone cortex was on the fistula hole, and the majority of the bone in the posterior wall of the external auditory canal was destroyed. Moreover, the upper as well as posterior tympanum and the mastoid process were fused into one large cavity with amber, soft, and easy-to-bleed organization; most of the malleus and incus parts were rotten; the stapes could not be seen; and the facial nerve bone canal and the semicircular canal bone were intact (Fig. 6a, b). Postoperative pure tone audiometry suggested sensorineural hearing loss in the left ear, and the average air conduction threshold was 100 dB; the right ear exhibited mixed deafness, and the average bone to hearing threshold was 30 dB, while the average air conduction threshold was 60 dB. On postoperative pathology examination (left ear), adipose tissue, smooth muscle and small blood vessels were observed in the tissue. This finding combined with immunohistochemistry was consistent with AML. The immunohistochemistry findings were as follows: SMA (+), desmin (−), CD34 (−), b-catenin (+), and Ki-67 index < 5% (Fig. 6c). The postoperative course was uneventful, and the patient was discharged after 9 days of anti-infection, haemostasis and fluid rehydration and did not seek further treatment. To date, there is no evidence that she has experienced recurrence.

## Discussion and conclusion

AML is the most common benign tumour of the kidney, most commonly found in middle-aged women, and is composed of perivascular epithelioid cells also known as perivascular epithelioid cell tumours (PEComas) [3, 7]. This is a mesenchymal tumour that is involved in the formation of hoof tissue, blood vessels, and the lymphatic system. However, AML is composed of different proportions of vascular cells, mature smooth muscle cells and fat cells. Some reports have described AML as a typical common tumour of the kidney, but currently, it is more commonly found in the liver; this tumour is also found in the ovaries, fallopian tubes, spermatic cords, palate and colon [8]. However, AML in the middle ear has not been previously reported. Considering that AML is rare, this study aimed to increase our understanding of AML outside of the kidney and liver.

### Clinical features

The clinical manifestations of AML lack specificity. There are no typical symptoms when the tumour is small. Patients may suddenly present local pain when the tumour bleeds internally. Haemorrhagic shock may occur when massive bleeding of the tumour occurs. In addition, purulent secretions found during physical examination may be caused by the destruction of surrounding tissues and infection by the tumour. Renal AML can be diagnosed by ultrasound, CT and MRI. However, AML in other locations is not common, and the nidus may be small, so the tumour cannot be clearly confirmed by CT and MRI. Further biopsy is needed for AML identification.

The patient in this case was further aggravated by coinfection with AML. She recovered very well after operation combined with anti-infection treatment.

Although this patient was coinfected and completely deaf in the left ear, there was no obvious destruction of the surrounding tissues by the tumour according to radiologic features in this case. However, if the tumour grows further, upward invasion may damage the tympanic canopy and skull base, leading to epidural abscess, purulent meningitis, and brain abscess; downward disruption may damage the jugular bulb, causing massive bleeding; disruption of the sigmoid sinus may result in thrombophlebitis or bleeding in the sigmoid sinus; anterior inferior disruption may damage the carotid artery tube, causing massive bleeding; posterior subperiosteal abscess and bezold abscess of the neck may be caused by bone defects and destruction of the mastoid tip or medial wall of the mastoid tip; and facial paralysis may be caused by disruption of the facial nerve bone canal and inflammation in the facial nerve.

The radiologic examination of this patient resulted in a misdiagnosis of AML as cholesteatoma. Preoperative identification remained difficult, but surgery is able to lead a satisfactory result in our case.

## Data Availability

The datasets used and analyzed during the current study are available from the corresponding author on reasonable request.

## Abbreviations

AML: Angiomyolipoma
CT: Computed tomography
MRI: Magnetic resonance imaging
PEComa: Perivascular epithelioid cell tumor

## References

[1] Flum AS, Hamoui N, Said MA, Yang XJ, Casalino DD, McGuire BB, Perry KT, Nadler RB (2016). Update on the diagnosis and management of renal angiomyolipoma. J Urol.

[2] Çalışkan S, Gümrükçü G, Özsoy E, Topaktas R, Öztürk M (2019). Renal angiomyolipoma. Rev Assoc Med Bras (1992).

[3] Park BK (2017). Renal angiomyolipoma: radiologic classification and imaging features according to the amount of fat. AJR Am J Roentgenol.

[4] Eble JN (1998). Angiomyolipoma of kidney. Semin Diagn Pathol.

[5] Bissler JJ, Kingswood JC, Radzikowska E, Zonnenberg BA, Frost M, Belousova E, Sauter M, Nonomura N, Brakemeier S, de Vries PJ (2013). Everolimus for angiomyolipoma associated with tuberous sclerosis complex or sporadic lymphangioleiomyomatosis (EXIST-2): a multicentre, randomised, double-blind, placebo-controlled trial. Lancet.

[6] Northrup H, Krueger DA (2013). Tuberous sclerosis complex diagnostic criteria update: recommendations of the 2012 Iinternational Tuberous Sclerosis Complex Consensus Conference. Pediatr Neurol.

[7] Lattanzi M, Deng FM, Chiriboga LA, Femia AN, Meehan SA, Iyer G, Voss MH, Sundatova Y, Huang WC, Balar AV (2018). Durable response to anti-PD-1 immunotherapy in epithelioid angiomyolipoma: a report on the successful treatment of a rare malignancy. J Immunother Cancer.

[8] Damaskos C, Garmpis N, Garmpi A, Nonni A, Sakellariou S, Margonis GA, Spartalis E, Schizas D, Andreatos N, Magkouti E (2017). Angiomyolipoma of the liver: a rare benign tumor treated with a laparoscopic approach for the first time. Vivo.

